# Beamcon III, a Linearity Measurement Instrument for Optical Detectors

**DOI:** 10.6028/jres.099.067

**Published:** 1994

**Authors:** Ambler Thompson, How-More Chen

**Affiliations:** National Institute of Standards and Technology, Gaithersburg, MD 20899-0001; Department of Electrical and Computer Engineering, University of Alabama in Huntsville, Huntsville, AL 35899

**Keywords:** beam addition method, linearity, optical radiation detectors, silicon photodiode

## Abstract

The design and operation of Beamcon III, the latest linearity measurement instrument using the beam addition method in the detector metrology program at the National Institute of Standards and Technology, is described. The primary improvements in this instrument are the reduction of stray radiation to extremely low levels by using three well-baffled chambers, a larger dynamic range, and an additional source entrance port. A polynomial response function is determined from the data obtained by this instrument using a least-squares method. The linearity of a silicon photodiode-amplifier detector system was determined to be within 0.054 % (2*σ* estimate) over nine decades of signal.

## 1. Introduction

The underlying assumption in the use of most detection methods for the measurement of optical radiation is that the detector output signal is directly proportional to the input radiation flux. This proportionality is defined as linearity and conversely the departure from proportionality is defined as nonlinearity. Optical radiation detectors are typically converters of optical power (or photon flux) to measurable electrical parameters, such as current, voltage, or pulse frequency. Therefore, in radiometric applications, the linearity of a detector's signal is not only a function of the efficiency of the physical radiation detector (transducer), but also of the peripheral electronics. A well known example of this is a photomultiplier/photon-counting system where significant nonlinearity is induced at high count rates by the dead time of the pulse counter. All detector systems, consisting of detectors and peripheral electronics, are characterized by some degree of nonlinearity. The exact positions of these nonlinear regions are functions of both the detector and the accompanying electronics and should be determined experimentally for measurements of the highest accuracy. There are essentially two practical choices when confronted by nonlinear behavior of a detector system: first, to view it as a performance limit, a maximum deviation from linearity over a given range of input; second, to correct the measured signal given the known nonlinearity of the detector. This latter solution presupposes that the detector's nonlinearity is well characterized and should be determined with the measurement electronics that are to be routinely used with the detector.

Concern about detector linearity and the development of instruments for its measurement has a long history at the National Institute of Standards and Technology, formerly the National Bureau of Standards (NBS). Sanders [1] reported in 1972 the results of photocell linearity measurements using a multi-aperture design. Also in 1972, Mielenz and Eckerle [2] utilized a double-aperture method to characterize the NBS Reference Spectrophotometer. In 1984 Saunders and Shumaker [3] reported measurements using an automated radiometric linearity tester. This instrument was called a beamconjoiner.

Based on this initial work, we now report on our latest generation of beamconjoiner technology, Beamcon III. This instrument was specifically designed to characterize radiometric detectors, particularly at low flux levels approaching the noise equivalent power (NEP) of the detector. To facilitate low level measurements, this instrument has three well-baffled chambers to reduce the stray radiation to extremely low levels. Other improvements are additional filters to give a larger dynamic range and a second source entrance port for a laser source or for source mixing. The data analysis algorithm of Beamcon III calculates the responsivity of the detector system assuming a polynomial relationship between the input flux and the output signal from the detector system. Beamcon III will serve as an automated instrument for routine detector linearity characterization and for determining the linearity of NIST's monochromator-based systems (e.g., spectroradiometers and spectrophotometers).

## 2. Description of Beamcon III and its Operation

**Fig. 1:**
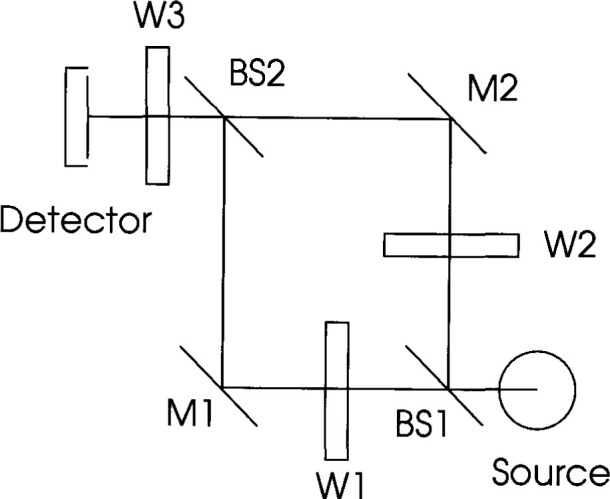
Schematic optical diagram of Beamcon III showing the primary optical elements. BS: beam splitter; W: filter wheel; and M: mirror.

A schematic diagram illustrating the technique of beamconjoining is shown in Fig. 1. The input beam from the source is split into two optical paths by the first beamsplitter BSl and variably attenuated by filters on either wheel Wl or W3. After reflection off mirrors Ml and M2, the two beams are combined by beamsplitting BS2 and attenuated again by a third filter on wheel W2 before falling on the detector. All filters are metallized neutral density filters with quartz substrates, and the filter wheels are tilted to preclude interreflection between filters. In addition the beamsplitters are wedged to prevent etaloning.

**Fig. 2:**
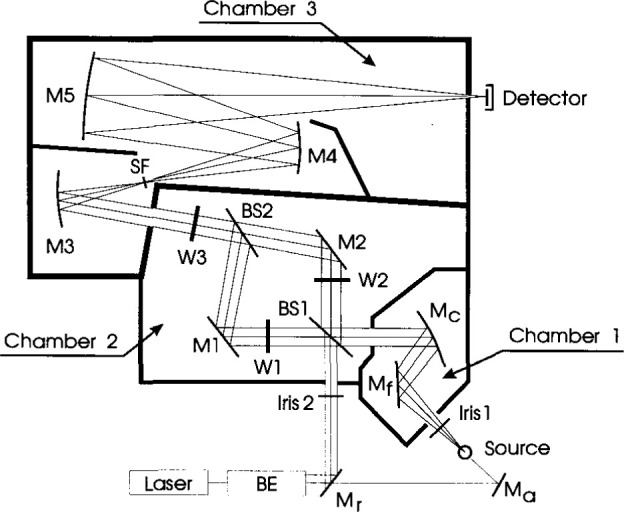
System layout of Beamcon III. Chamber 1 collimates the beam from the source. Chamber 2 splits the beam into two paths and recombines it. Chamber 3 has a spatial filter SF to eliminate stray light and focuses the beam onto the detector.

A complete schematic diagram of Beamcon III, including all ports, optics, and chambers, is shown in Fig. 2. Beamcon III provides four basic advantages over the previous technology: 1) each chamber is optically isolated from the next chamber except for a 3 inch diameter throughput port, thereby reducing scattered radiation; 2) there are two source input ports for both laser and broadband sources either singly or concurrently for source mixing; 3) a steel base plate allows for magnetic mounting of additional optical components; 4) an external translation stage on the broadband input port can vary the numerical aperture and colllmate the source beam. A microcomputer controls the positions of the filter wheels and records the signal from the detector.

An incoherent light source (Source, Fig. 2), either a quartz halogen or arc lamp, is placed on a translation stage in front of the input port to Chamber 1. The input power of the source beam is varied either by changing the aperture of the iris diaphragm (Iris 1) or by translating the Source, or both. The input beam in Chamber 1 is folded by a flat mirror (M_f_) and collimated by a concave spherical mirror (M_c_), which projects the beam through the input port of Chamber 2. For narrowband operation with an incoherent light source, an interference filter can be placed at the input port of Chamber 2. The first beamsplitter (BSl) yields two separate beams which are independently attenuated by two filter wheels (Wl) or (W3). These filter wheels contain four neutral density filters with differing optical densities. The two beams are folded by mirrors (Ml) or (M2) and recombined into a single beam by beamsplitter (BS2). This beam is further attenuated by passing through a third filter wheel (W2) containing five different neutral density filters. The beam then passes into Chamber 3, which contains a set of concave mirrors (M3 and M4). A spatial filter (SF) at the focal point between the two mirrors filters out scattered and diffracted light, resulting in a uniform output field at the detector. A concave mirror (M5) focuses the beam onto the detector. Translational shifting of M5 controls the image plane of the instrument and varies the irradiance at the detector. The detector is generally overfilled to prevent artifacts due to different cut-off edges in the optical path (optical stop variation) and to the local nonuniformity of the detector. In addition environmental parameters such as humidity and temperature are constantly monitored at the detector. A monochromator can also be attached at the exit port to select a wavelength for narrowband operation.

Beamcon III has a second input port into Chamber 2, which can be used singly or in concert with the beam from Chamber 1. Generally the input port of Chamber 2 is used for sources such as He- Ne or Ar^+^ lasers. A beam expander BE is used to minimize scattering losses and filter transmittance nonuniformity. The laser can also be used to align both optical paths by removing the reflecting mirror (M_r_) and inserting the mirror (M_a_) to guide the laser beam through the center of the lamp filament and of every optical element. The mixed source method has not been attempted as yet, but is planned in future studies, as it has advantages for studying differential spectral detector nonlinearities [4].

Stepping motors control the filter wheel positions, each with 400 steps/revolution. A tolerable error is one step, therefore the error in filter position is 0.9°. The space between two filters on each wheel acts as a shutter position for a dark signal measurement. Thus, the total number of filter combinations is 150, and a full 150 measurements is called a pass. Of these, 30 are dark signal measurements: 5 when both Wl and W3 are shuttered and 25 when W2 is shuttered. The remaining 120 filter combinations, yielding 120 response signal measurements, are randomized in each pass to prevent hysteresis. The dark signal/filter combinations are randomized and inserted after every five measurements. Each dark signal is used to correct the succeeding five response signal measurements. Generally, five passes are taken and averaged to reduce the random error and to collect information about possible source, filter, and detector drift. The maximum throughput is 7 % at a wavelength of 632.8 nm, and the total attenuation factor is about 3000 against a light source.

The output beam is partially vertically polarized for an unpolarized source since each tilted surface preferentially reflects the vertical component of the incident beam. The ratio of the polarization in the vertical direction to that in the horizontal direction is 1.17. Thus, it is critical to keep the polarization direction of a source (e.g., laser) constant in order to eliminate this polarization preference.

## 3. Polynomial Response Function Fitting

The principle of operation of Beamcon III is the beam addition method, in which different filter wheel combinations result in different fluxes at the detector. This method assumes that the flux from the source remains constant during all measurements and that the output flux at the detector is the sum of the two fluxes along the two different paths.

The final flux *ϕ* at the detector is the sum of the fluxes *ϕ*_1_ and *ϕ*_2_ from the two paths. Indexing the fluxes by the filter combination,

$$\phi \left(i,j,k\right)=\phi_{1}\left(i,k\right)+\phi_{2}\left(j,k\right),$$ (1)

where *i* equals 0 to 4 designates the filter on wheel W1, *j* equals 0 to 4 the filter on W3, and *k* equals 0 to 5 the filter on W2. In each case, an index value of 0 corresponds to an opaque, shuttered position. For a single path, there are 30 possible filter combinations. Of these, no flux is transmitted for 10 combinations. Thus, each path has 20 filter combinations that result in a response signal.

Since the detector is assumed to be nearly linear, it is appropriate and convenient to assume an *n*-th order polynomial response function for the detector [3], so that

$$\phi \left(i,j,k\right)=r_{0}+r_{1}s\left(i,j,k\right)+r_{2}s^{2}\left(i,j,k\right)+\ldots +r_{n}s^{n}\left(i,j,k\right),$$ (2)

where *s*(*i,j,k*) is the measured response signal, corrected by the dark signal and indexed by the filter combination, and *r*_0_, *r*_1_, *r*_2_, …,*r_n_* are the response function coefficients. The coefficient *r*_1_ is set equal to one, both because the detector is assumed to be nearly linear and to normalize the flux to the same units as the signal.

Combining Eqs. (1) and (2), with *r*_1_ = 1, yields

$$\begin{array}{c} \phi_{1}\left(i,k\right)+\phi_{2}\left(j,k\right)=r_{0}+s\left(i,j,k\right)+r_{2}s^{2}\left(i,j,k\right)\\ +\ldots +r_{n}s^{n}\left(i,j,k\right). \end{array}$$ (3)

There are 120 expressions of the form of Eq. (3) from the 120 distinct response signals measured in each pass, with 40+*n* unknown variables from the 20 filter combinations along each path and the *n* response function coefficients. The fluxes of shuttered filter combinations are set equal to zero. These 120 expressions are solved using the linear least-squares technique to determine the values of the unknowns. While the values of the polynomial response function coefficients are the primary goal of the measurements, the values of the fluxes are useful for monitoring the stability of the filters.

Solving for both the fluxes along the paths and the response function coefficients provides two ways in which to determine the total flux at the detector, using Eqs. (1) and (2), and therefore serves to verify the results. A measurement run yields 120 residuals between the total fluxes calculated in these two ways. If there is a slope in a time-ordered plot of these residuals, then either dark signal or source intensity variations likely exist. Also, the standard deviation of these residuals indicates the noise level present during the measurement, either from the source, filters, or detector. For most detectors, a second-order polynomial accurately fits the data. However, if the detector is highly nonlinear, the power of the polynomial is increased until the residuals are normally distributed about zero. If the response function coefficients remain unreasonable, then either a polynomial is not an appropriate fitting function or the detector and its associated electronics are not functioning properly.

The algorithm for determining the 40+*n* unknown variables from a complete measurement was extensively tested using synthetic data sets with known response function coefficients for secondorder polynomials. It was also tested using synthetic data sets corresponding to two cases of nonlinear behavior, supraresponsivity and saturation at high flux levels [5]. The algorithm was successful in fitting these two behaviors with a third- and a sixth-order polynomial, respectively.

## 4. Linearity Measurement of a Silicon Photodiode

Beamcon III was used to determine the linearity of a silicon photodiode-amplifier detector system designed and constructed at NIST for multi-decade performance and described previously [6]. The linearity was determined for amplifier gains from 10^4^ to 10^10^. The broadband source was a 1000 W tungsten-halogen lamp operated at a constant current of 7.6 A. In order to cover the entire signal range of the detector system without changing the spectral distribution of the source, the flux from the lamp was varied either by changing the numerical aperture of Iris 1 in Fig. 2 or by placing neutral density filters in Chamber 1, or both.

**Fig. 3:**
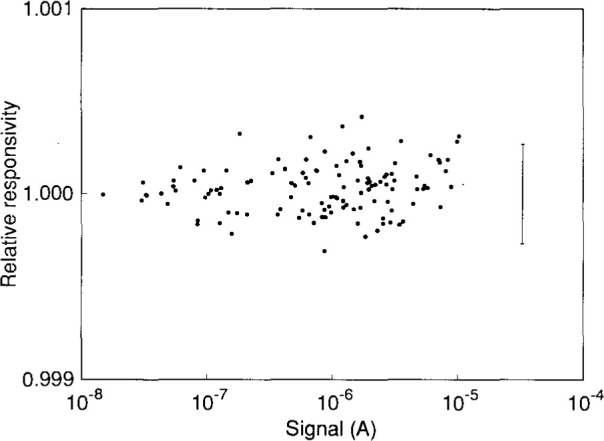
Relative responsivity as a function of signal for a silicon diode-amplifier detector system at a gain of l0^6^. The error bar indicates the 2*σ* estimated uncertainty of the relative responsivity.

The relative responsivity of a detector system indicates deviations from linearity. Once the response function coefficients have been determined, the total normalized flux for a given signal is given by the response function [the right-hand-side of Eq. (3)]. If the detector system is linear, the total normalized flux is simply the signal. Therefore, the relative responsivity at a given signal is the response function divided by the signal. The results from one measurement of the linearity of the detector system at a gain of 10^6^ is shown is Fig. 3, where the relative responsivity is plotted as a function of signal. The response function was a second-order polynomial with coefficients *r*_0_=−5.53×10^−9^ and *r*_2_=−1.56×10^−5^ Over the three decades of signal measured at this gain, the detector system is linear to within 0.027 % (2*σ*-estimate).

**Fig. 4:**
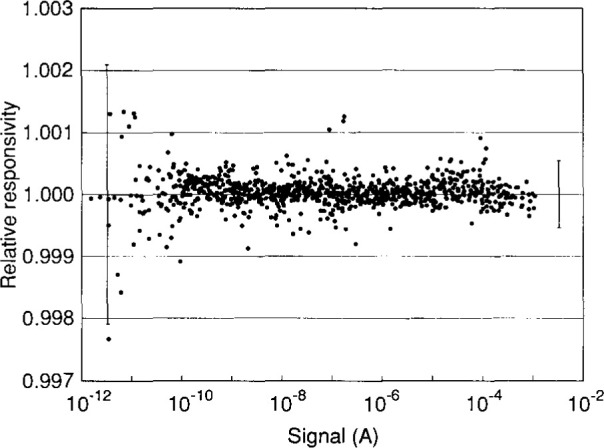
Relative responsivity as a function of signal for a silicon diode-amplifier detector system over its entire signal range of nine decades. The error bars indicate the estimated 2*σ* uncertainty of the relative responsivity for signals less than 10^11^ A (on the left) and over the entire range of signal (on the right).

The maximum signal for the detector system, determined by the source and throughput of Beamcon III, was about 10^−3^ A. At a bandwidth of 0.67 Hz, the NEP of the detector system resulted in a minimum measurable signal of about 10^−12^ A. In order to characterize the detector system between these limits, linearity measurements were performed at all seven amplifier gain ranges. A second-order polynomial response function was calculated for each range, and the relative responsivity over nine decades of signal is shown in Fig. 4. The detector system is linear to within 0.054 % (2*σ* estimate) over the entire range, and to within 0.209 % (2*σ*-estimate) for currents less than 10^−11^ A. Thus, Beamcon III was able to determine the linearity of this detector system for signals approaching its NEP.

## 5. Conclusions

The design and operation of our latest generation of beamconjoining technology, Beamcon **III**, was detailed. Using this instrument, the linearity of a silicon photodiode-amplifier detector system was determined over nine decades of signal, approaching its NEP at the smallest signals. Beamcon III will permit both routine calibration of detector systems and research into detector-amplifier effects on nonlinearity. Future studies on the linearity of solid state detector-amplifier systems, photomultiplier photon-counting systems, and multichannel detectors (i.e., diode arrays and charge coupled devices) are planned, as well as on the capabilities of Beamcon III for source mixing.
